# Whatever Works: A Systematic User-Centered Training Protocol to Optimize Brain-Computer Interfacing Individually

**DOI:** 10.1371/journal.pone.0076214

**Published:** 2013-09-23

**Authors:** Elisabeth V. C. Friedrich, Christa Neuper, Reinhold Scherer

**Affiliations:** 1 Department of Psychology, University of Graz, Graz, Austria; 2 Institute for Knowledge Discovery, Graz University of Technology, Graz, Austria; University Medical Center Groningen UMCG, Netherlands

## Abstract

This study implemented a systematic user-centered training protocol for a 4-class brain-computer interface (BCI). The goal was to optimize the BCI individually in order to achieve high performance within few sessions for all users. Eight able-bodied volunteers, who were initially naïve to the use of a BCI, participated in 10 sessions over a period of about 5 weeks. In an initial screening session, users were asked to perform the following seven mental tasks while multi-channel EEG was recorded: mental rotation, word association, auditory imagery, mental subtraction, spatial navigation, motor imagery of the left hand and motor imagery of both feet. Out of these seven mental tasks, the best 4-class combination as well as most reactive frequency band (between 8-30 Hz) was selected individually for online control. Classification was based on common spatial patterns and Fisher’s linear discriminant analysis. The number and time of classifier updates varied individually. Selection speed was increased by reducing trial length. To minimize differences in brain activity between sessions with and without feedback, sham feedback was provided in the screening and calibration runs in which usually no real-time feedback is shown. Selected task combinations and frequency ranges differed between users. The tasks that were included in the 4-class combination most often were (1) motor imagery of the left hand (2), one brain-teaser task (word association or mental subtraction) (3), mental rotation task and (4) one more dynamic imagery task (auditory imagery, spatial navigation, imagery of the feet). Participants achieved mean performances over sessions of 44-84% and peak performances in single-sessions of 58-93% in this user-centered 4-class BCI protocol. This protocol is highly adjustable to individual users and thus could increase the percentage of users who can gain and maintain BCI control. A high priority for future work is to examine this protocol with severely disabled users.

## Introduction

A brain-computer interface (BCI) translates physiological brain signals into an output that reflects the user’s intent. It can provide severely motor-impaired users with a new, non-muscular channel for communication and control which may be their only possibility to interact with the external world [1]. There is also growing attention to non-medical applications, such as using BCI technology for gaming [2,3] or art [4], as well as for cognitive monitoring of brain activity [5,6].

One way to implement a BCI involves non-invasively recording the rhythmic activity of the brain’s electrophysiological signals by electroencephalography (EEG) and detecting the amplitude changes (event-related (de)synchronization, ERD/S [7]) that users voluntarily produce. In most studies, able-bodied as well as disabled participants used motor imagery tasks (i.e. the kinesthetic mental imagination of movements) to induce characteristic ERD/S patterns (e.g. [8–11]). However, non-motor tasks such as mental subtraction or mental cube rotation can also be used for BCI control [12–18]. We believe that the combined use of mental tasks which are intrinsically generated in spatially distinct cortical areas (e.g. verbal, motor, spatial tasks) will better account for individual differences and be most valuable for individuals with brain injury and disrupted cortical networks [13,19–22]. However, recent studies including able-bodied as well as disabled individuals revealed huge individual differences in best task combinations [18,22].

In our previous study [23], fourteen able-bodied users were trained for 10 sessions plus a follow-up session to control a 4-class BCI. Every participant performed the same mental tasks (i.e. word association, mental subtraction, spatial navigation, and motor imagery task) for 7 s (i.e. imagery period) in each trial while EEG was recorded. For online control, the EEG was band pass filtered within a predetermined frequency band, and ERD/S patterns were extracted from the last 6 s of the imagery period and used for classification and feedback presentation. Classifier updates were made following a predefined protocol. Continuous feedback was provided to the user which indicated in real-time which task was selected by the classifier at that very moment. The mean performance stayed stable in the follow-up session after months without training or classifier adjustments. However, general performance was rather low for effective BCI control in daily life and did not increase with training but was rather unstable and unbalanced between tasks. That means that a user might have achieved high accuracies in the word association and low accuracies in the mental subtraction task in one session, while showing the reverse in the next session, for example.

To counter these effects, we implemented a new BCI protocol, which was more user-centered and designed to make individual adjustments and optimizations possible. A user-centered design adjusts the protocol to the user rather than imposing a predefined protocol on the user. Zickler et al. recently adapted a user-centered developmental process from assistive technology to BCI research [24]. In our present study, we only addressed one aspect of such a user-centered approach, namely providing an improved protocol (i.e. design solution) to meet user requirements for more accurate and faster BCI control [25].

Typically, all participants use the same predefined task combinations for online control (e.g. [23,26]). Only in few studies, the task combinations were selected individually for 2-class or 3-class BCI control [14,19]. In the present study, we recorded one screening session with seven mental tasks which were chosen according to our gained expertise in prior research [18,22,23,27,28]. Out of these seven mental tasks, the best 4-class combination was selected individually for online control.

Discriminatory information extracted by common spatial patterns within a broad frequency range of 8-30 Hz achieved reasonably high classification performances in the studies of Müller-Gerking et al. [29] and Ramoser et al. [30] without the need of user-specific optimization. For this reason we used this predetermined frequency range in our previous study [23]. In a more recent study, Blankertz et al. [31] implemented individually optimized frequency ranges for every participant to discriminate two motor imagery tasks. In the present study, at the cost of higher computational time for optimization, we adapted this promising approach to the classification of motor and non-motor tasks in a 4-class BCI and selected the best frequency range individually.

To overcome the problem of unstable and unbalanced performance between tasks and sessions, which was a major problem in our previous study [23], we used the geometric rather than the arithmetic mean for the selection of the best task combination and frequency range. Additionally, we adapted the classifier bias (i.e. calibration) at the beginning of each session as suggested in the literature [32,33]. Moreover, the number and time of classifier updates between sessions varied individually.

We hypothesize that individual optimization of classification and individual selection of task combination and frequency range will enhance performance and robustness of BCI control. Our first hypothesis H1 proposes that all users will perform significantly above chance when following the proposed training paradigm. The second hypothesis H2 suggests that performance will increase with practice.

Previous findings showed that classification performance of different mental tasks changed over time within one imagery period [34]. The evolution of brain patterns over time contributes to these changes in classification accuracy. Friedrich et al. attributed the temporal changes of ERD/S patterns during the imagery period of various mental tasks to the complex nature of these tasks as they all involve different sub-tasks, processes and brain structures that influence each other [28]. Thus, it may be difficult for the users to maintain a specific brain pattern for a long period of time. This is required by the selected signal processing method which is designed to characterize a time-invariant pattern of sustained brain activity. In order to make selections easier for the user as well as faster, we considered the temporal component of classification [23,34,35]. Reducing the length of the imagery period (and thus the whole trial) can enhance communication and control speed. Therefore, we restricted the imagery as well as the classification period in the present study to 6 s and 4 s, respectively, which is shorter than in previous cue-based protocols including non-motor tasks [15,16,23]. Thus, our third hypothesis H3 is that classification time can be reduced for motor and non-motor tasks in a 4-class BCI without a decrease in performance.

Typically, the classifier is built and adjusted on the data recorded in the screening and calibration runs in which no continuous visual feedback is provided to the users. This classifier is then used in runs with real-time feedback. However, several studies reported significant differences in brain activity between sessions with and without continuous feedback [10,23,32,36]. Therefore, we presented sham feedback in the screening and calibration runs that had the same characteristics as the continuous real-time feedback in the feedback sessions. Accordingly, the classifier was built on brain activity that was more similar to the brain activity demonstrated during online control. Users were made aware that the sham feedback was not related to their brain activity or performance. As a result, differences between sessions with and without feedback that are originated by the visual information presented rather than to the processing of the meaning of the feedback are reduced.

Continuous real-time feedback was only provided to the user if the classifier detected the correct (i.e. indicated by the cue) task in the feedback sessions. Otherwise, no continuous feedback was displayed. This was done to help participants to focus on the indicated task and avoid speculations about why the classifier might have chosen this rather than another task. In order to mimic a reasonably high positive feedback, sham feedback was presented in approximately 70% of the trials for each class in the screening and calibration runs. In doing so, user received positive feedback in the majority of cases, however, also no feedback that was indicative of misclassification. This leads to our fourth hypothesis H4, which proposes that differences in brain activity (i.e. ERD/S) between the screening and the first feedback session can be reduced due to sham feedback.

To summarize, in this study we tested the following hypotheses:

H1: All users achieve accuracies that are significantly above chance level within few training sessions by using the proposed user-centered BCI protocol.

H2: Performance increases over training sessions by individual optimization of control strategies (i.e. mental tasks), frequency range and classifier updates.

H3: The classification periods can be made shorter without compromising accuracy.

H4: Sham feedback reduces differences in the ERD/S patterns between sessions with (i.e. feedback sessions) and without feedback (i.e. screening session).

## Methods

### 1: Ethics Statement

The work has been conducted in accordance with the relevant guidelines for ethical research according to the Declaration of Helsinki and has been approved by the ethical committee of the University of Graz. All participants gave their written informed consent to the study.

### 2: Participants

This study included 8 participants who were initially naïve to the use of a BCI and the tasks. The 3 men and 5 women aged between 20-36 years (mean age = 25), had no medical diseases and were all right-handed. Each volunteer participated in one screening session (i.e. session 1) and then in 9 feedback sessions (i.e. sessions 2-10) over a period of 4-6 weeks.

### 3: EEG recordings

The EEG was recorded from 29 sintered silver-silver chloride (Ag/AgCl) ring electrodes (EASY CAP, Hersching, Germany) in the standard positions according to the extended 10–20 system (F3, Fz, F4, FT7, FC5, FC3, FCz, FC4, FC6, FT8, C5, C3, C1, Cz, C2, C4, C6, CP3, CPz, CP4, P5, P3, P1, Pz, P2, P4, P6, PO7, PO8) and referenced to the left and grounded at the right mastoid. Additionally, electrooculogram signals for vertical and horizontal eye movements were recorded. The electrode impedance was kept below 5 kOhm. The EEG was amplified by g.USBamps (Guger Technologies, Graz, Austria) and the system software was implemented in a MATLAB-based Simulink model. The data were filtered (0.5-100 Hz) and sampled at 256 Hz. During the EEG recordings, the participants were sitting in a comfortable chair in front of a 17″ monitor at a distance of about 1.1 m in an electrically shielded recording room.

For the calculation of the classifier as well as the ERD/S analyses, the EEG data were inspected visually and trials contaminated with muscle activity were removed. Additionally, the data were corrected for electrooculogram artifacts via the method described in Gratton et al 1983 [37] and implemented in the Brain Vision Analyzer (Brain Products, Gilching, Germany).

### 4: Mental tasks

We used two brain-teaser tasks (i.e. tasks that require the user to solve a specific mental problem, such as word association and mental subtraction), two dynamic motor imagery tasks (motor imagery of the left hand and of both feet), two dynamic non-motor imagery tasks (auditory imagery and spatial navigation) and a dynamic visualization task (mental rotation).

1. Word association (WORD): generate as many words as possible in your mother tongue (i.e. German) that begin with a presented letter (e.g. P_ = price, etc.), end with a presented letter (e.g. _P = map, etc.) or have the presented letter in the middle of the word (e.g. _P_ = adaptation, etc.);
2. Mental subtraction (SUB): perform successive elementary subtractions by a presented fixed number (e.g. 105-14 = 91, 91-14 = 77, etc.);
3. Motor imagery of the left hand (HAND): imagine kinesthetically repetitive self-paced movements of the own left hand squeezing a ball without any actual movement;
4. Motor imagery of the feet (FEET): imagine kinesthetically repetitive self-paced movements of both feet without any actual movement;
5. Auditory imagery (AUD): imagine listening to a familiar tune without articulating the words but rather focusing only on the melody;
6. Spatial navigation (NAV): imagine navigating through a familiar house or flat from room to room, focusing on orientation rather than on movement;
7. Mental rotation (ROT): visualize a 3-dimensional L-shaped figure to rotate in the 3-dimensional space.

### 5: Experimental paradigm

**Figure 1:**
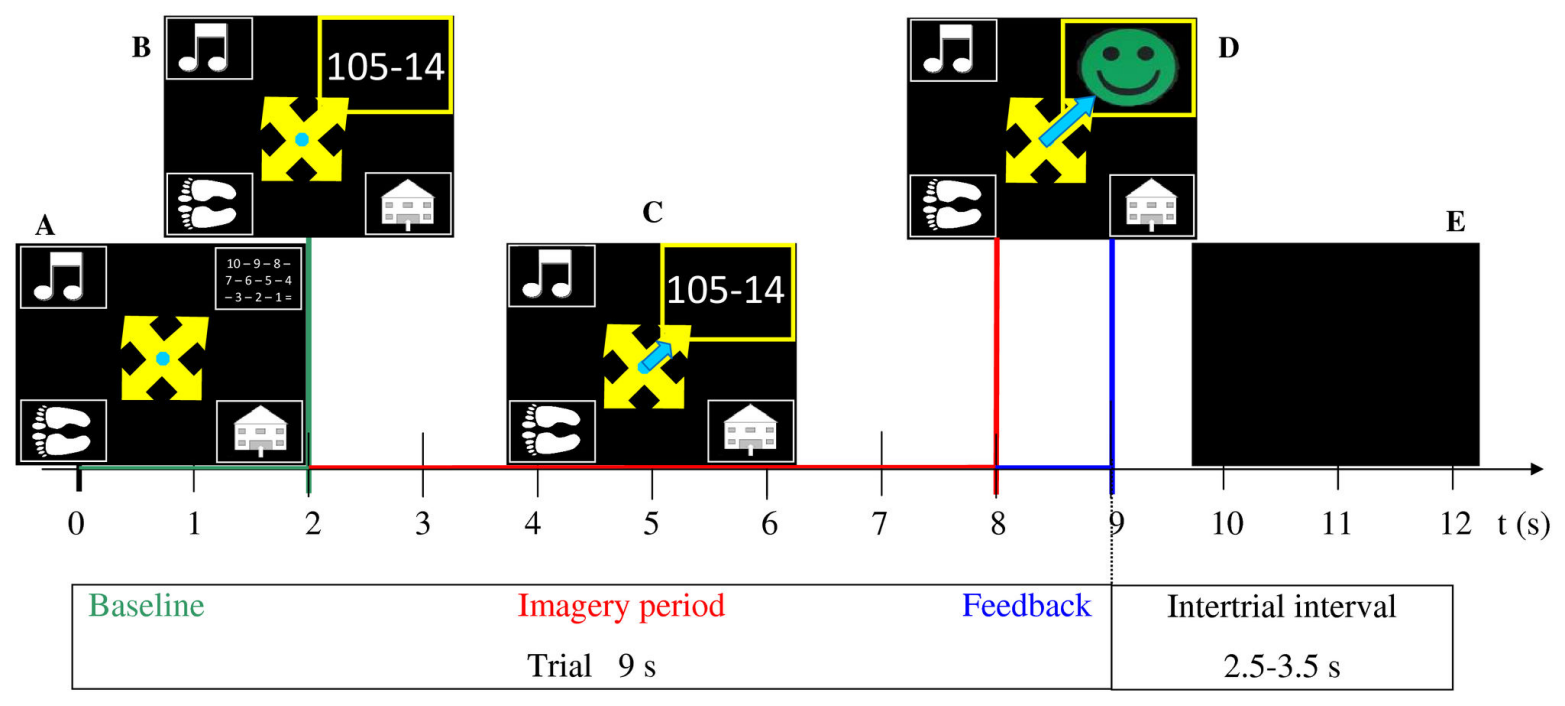
Experimental paradigm of one trial. (A) At t = 0 s, four symbols representing four tasks and a fixation cross were presented on the screen (baseline). (B) At t = 2 s, one symbol was randomly highlighted and in case of the word association or mental subtraction task, an initial letter or the specific subtraction, respectively, was presented. (C) Users were asked to perform the indicated task for 6 s (i.e. imagery period from t = 2-8 s) while a blue bar graph was provided from t = 3-8 s (i.e. feedback period). In the feedback sessions, the blue bar graph represented the real-time feedback and was only shown if the classifier detected the correct task (i.e. from the cue indicated task) at this very moment. In the screening or calibration run, the blue bar graph was shown as sham feedback independently from performance in about 70% of the trials (see methods Sections 5 and 6). The relevant time for classification was from t = 4-8 s (i.e. classification period). (D) At t = 8 s, discrete feedback in form of a smiley was shown for 1 s if the given task was detected correctly (see methods section 6). If this was not the case, no feedback was displayed. (E) At t = 9 s, the screen remained blank for 2.5-3.5 s, before the next trial started.

Each session was divided in 6 runs and lasted about 2 h including the instructions, EEG montage, self-reports (not analyzed in this study) and the EEG recording (54 min in the screening, 48 min in the feedback sessions) with breaks between the runs. In the screening session, each run contained 49 trials (7 trials x 7 tasks), thus in total 42 trials per mental task (7 trials x 6 runs) were recorded. In the feedback sessions, each run contained 40 trials (10 trials x 4 tasks), thus in total 60 trials per mental task (10 trials x 6 runs) were recorded. The temporal structure of one trial is illustrated in Figure 1.

In the screening session, the combination of symbols - which represented the mental tasks - changed on the screen in every trial (see Figure 1). Thus, four out of the 7 mental tasks were randomly indicated on the 4 positions of the screen. Then, one of the four symbols was randomly highlighted (i.e. cue) in every trial and the user performed the indicated task for 6 s while staying as relaxed and motionless as possible. The sequence was pseudo-randomized to ensure that each class and each position of the screen was counterbalanced. The sham visual feedback was shown in 5 out of 7 trials per task and run. This sham feedback was displayed in the form of a bar graph which was identical to the one used for the continuous real-time feedback (see Figure 1).

After the screening, the 4-class combination with the highest offline accuracy was selected individually and was used in sessions 2-10 for online control (see methods section 6). The four symbols representing the four chosen tasks were randomly displayed in one of the four positions on the screen in every trial. This randomization ensured that the position of the cue on the screen had no impact on the elicited brain patterns. In every trial, one of the four symbols was then randomly highlighted and the user performed the indicated task for 6 s while the EEG was recorded (see Figure 1). This randomization was performed to minimize brain pre-activation due to expectation. The data of the first run (i.e. 10 trials per task) were used for calibration of the classifier (i.e. calibration run, see methods section 6). In the calibration run, the same sham feedback as in the screening session was shown in 7 out of 10 trials per task. In the following 5 runs, continuous real-time feedback was provided to the users (see Figure 1 and methods section 6). Furthermore, discrete feedback in the form of a smiley was provided at the end of a trial each time the selected mental task was classified correctly more often during the classification period (t = 4-8 s after trial onset) than the other tasks (see Figure 1 and methods section 6).

### 6: Signal processing and online classification

The common spatial patterns (CSP; [29–31]) method was used to design spatial filters that best characterized the different mental tasks in the EEG. The logarithm of the normalized variance for the filters with the two highest eigenvalues were computed and discriminated by means of Fisher’s linear discriminant analysis (LDA) classifiers [38]. Standard CSP and LDA methods were designed for 2-class problems [30,38]. For 4-class classification, six individual LDAs - one for each possible combination of pairs of mental tasks - were trained and majority voting was applied. CSP filters were computed for each subject from 5-s EEG segments extracted after cue presentation from t = 2.75 s to 7.75 s [39].

Only artifact-free EEG trials (see methods section 3) from one session (42 trials per task in the screening and 60 trials per task in the feedback sessions) were included in the analysis. The first classifier was calculated for each participant from data of the screening session. Out of all possible 4-class combinations, the combination with the highest geometric mean accuracy was selected for the 8-30 Hz frequency band. The geometric mean, defined as the N-th root of the product of N numbers, privileges combinations in which the accuracy is balanced and the variance reduced across tasks. The classifier generalization was estimated by using a 10-times 10-fold cross validation procedure. For each fold a BCI simulation (off-line simulation of on-line experiments; details on on-line processing are presented later in the text) was computed. For each discrete time point within the imagery period the geometric mean of the BCI simulation accuracies of the six individual LDAs was computed. The maximum geometric mean value was then chosen for the selection of the mental tasks. For the selected 4-class combination, an exhaustive search was performed to find the single frequency band that could discriminate best between the four tasks (i.e. achieved highest geometric mean).

In the following sessions, real-time feedback was provided to the users. For each feedback session, the offline-computed classifier was calibrated with data from the first run (i.e. first 10 trials per task). By calibration we mean that the distance of each of the original six LDA hyperplanes to the origin of axis was computed from the first 10 trials of each class to better fit the distribution of the new data. This corrects for day-to-day variability within the participant and the EEG montage (i.e. electrode impedance). LDA weights, i.e., the orientation of the LDA hyperplanes, for the CSP patterns remained unchanged [40].

During on-line experiments the log normalized variance of the spatially filtered EEG time series was computed from 2-s segments. Signal processing was performed on a sample-by-sample basis. Continuous real-time feedback was given in the form of a bar graph from t = 3-8 s after trial onset (i.e. feedback period, see Figure 1) only if the classifier detected the correct (i.e. from the cue indicated) task at that very moment. If the correct task was not detected, no continuous feedback (i.e. bar with zero length) was shown. Discrete feedback (i.e. a reward signal in the form of a smiley, see Figure 1) was provided at the end of a trial each time the selected mental task was classified correctly more often during the classification period (t = 4-8 s after trial onset) than the other tasks. To get a smooth and stable feedback control signal at discrete times t_i_, the classification results of the past second ([t_i_ -1 t_i_] s interval) were normalized, i.e., the length of the feedback bar was computed by counting the samples that were correctly classified and dividing the sum by the sampling rate (256 Hz).

**Figure 2:**
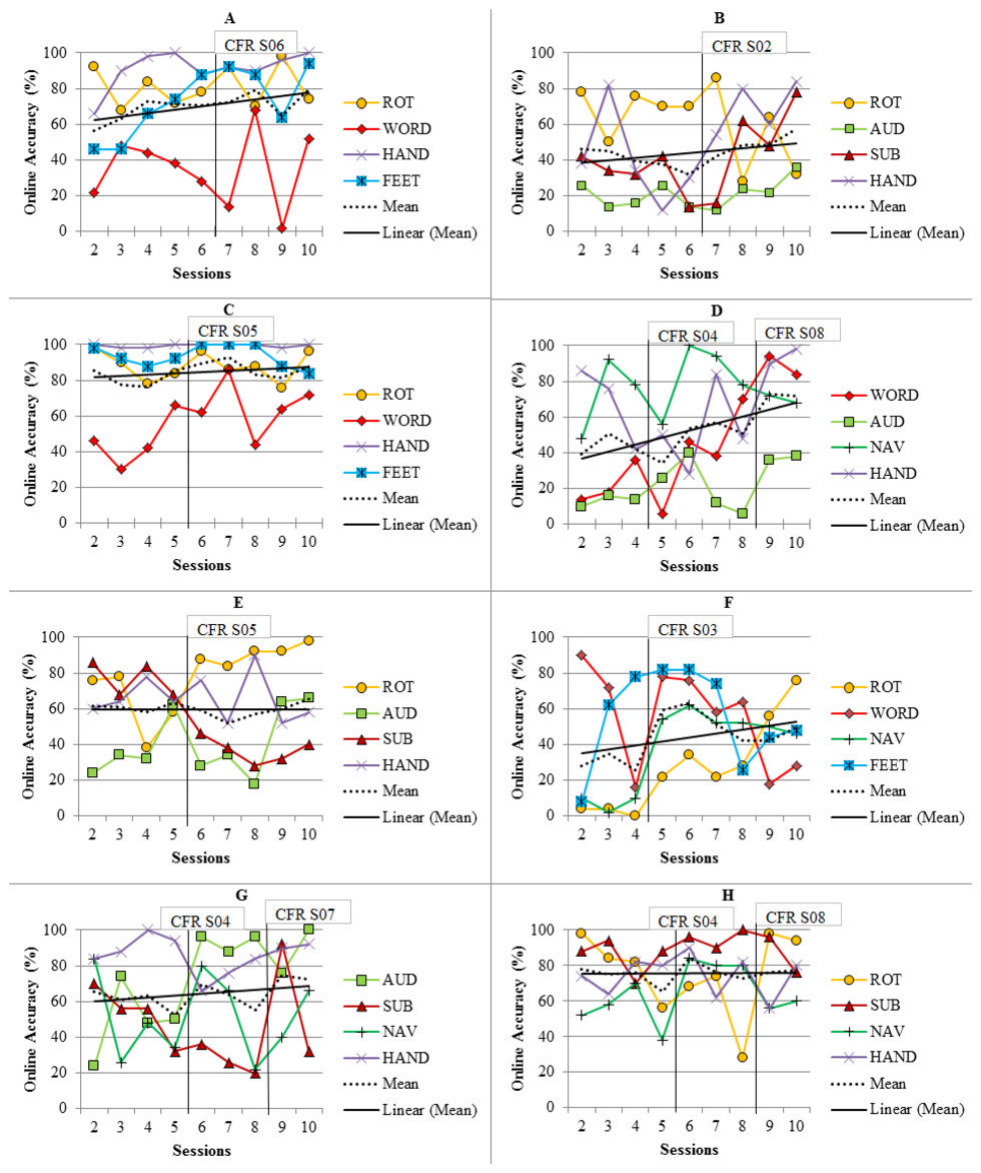
Online performance and classifier updates for all users (A-H). The vertical lines indicate the time of the updates and the labels indicate the sessions from which the classifier was recalculated (e.g. CFR S06: classifier was calculated with data recorded in session 6).

The number and time of updates as well as the session from which the data was used for the recalculation of the classifier varied individually (see Table 1 and Figure 2). With every classifier update, also the optimization of the frequency range was recalculated. The update criterion was based on the individual performance. An update was performed when offline classification and BCI simulation indicated that a performance improvement could be expected with a new classifier. However, increase of performance was only defined by descriptive values. As we did not follow statistical or predefined rules, we evaluate in the results section if the performed updates led to increased performance.

**Table 1:** Selected task combinations, frequency range and online performance per user.

User	Task combinations with mean online performance in % (SE)							Frequency range [Hz]			Online Performance [%]			
	ROT	WORD	AUD	SUB	NAV	HAND	FEET	Screen	Upd1	Upd2	Mean(SE)		Peak	Session
A	81 (4)	35 (7)				91 (3)	73 (6)	8-30	11-26	-	70 (4)	80		10
B	62 (7)		21 (3)	41 (7)		53 (9)		9-17	8-15	-	44 (4)	58		10
C	88 (3)	57 (6)				99(0)	94 (2)	8-19	10-25	-	84 (3)	93		7
D		45 (10)	22 (4)		76 (6)	67 (8)		9-26	9-14	9-15	53 (5)	73		9
E	78 (6)		40 (6)	54 (7)		66 (4)		9-15	8-16	-	60 (4)	66		10
F	27 (8)	56 (9)			38 (8)		56 (9)	8-30	8-13	-	44 (5)	64		6
G			72 (9)	47 (8)	52 (8)	86 (3)		8-20	11-23	10-25	64 (4)	75		9
H	76 (8)			89 (3)	64 (5)	74 (4)		10-30	9-20	11-30	76 (3)	85		6

The columns 2-8 indicate the mean online performance over all sessions with the standard error (SE) for the task combinations, which were selected for BCI control. For the selected task combination, the classifier was computed for the frequency range with the highest geometric mean between 8-30 Hz and a bandwidth between 2-22 Hz from the screening data (see column “Screen”). After the sessions 4-6, the classifier and thus the optimization of frequency range were recalculated (see column “Upd1”). For user D, G and H, the frequency range optimization and the classifier were updated another time (see column “Upd2”). The mean online performance averaged over sessions and tasks with standard error (SE) is indicated in the column 12. Additionally, the peak performance and in which single-session it was achieved is presented.

### 7: ERD/S and statistical analyses

For the calculation of the ERD/S patterns, the artifact-free EEG was re-referenced according to the common average reference method (CAR [41]). The ERD/S (i.e. the percentage power decrease/increase in relation to the reference interval) values of the imagery period (t = 2-8 s after trial onset) were calculated relative to the baseline (t = 1-2 s after trial onset) for 4-40 Hz frequency bands with a bandwidth of 2 Hz [42]. The bootstrap significance test was applied with an alpha level of 5%.

For the statistical analyses, normal distribution of the data was approved by the Kolmogorov-Smirnov test. Statistical comparisons were realized with paired-t-tests or with repeated-measurement analyses of variance (ANOVA). The Greenhouse–Geisser epsilon was taken for correction, as the assumption of sphericity was not met for the ANOVA. The post hoc tests were conducted with the Newman-Keuls test. Additionally, a linear regression was calculated to evaluate if performance increased linearly over sessions.

## Results

### H1. Online performance

The results confirmed that selected task combinations and frequency ranges differed between users (see Table 1). We found, however, that for each user one motor task (hand or feet or both) and one brain-teaser task (word association or mental subtraction but never both) was included in the task combination. The mental rotation task was also included in the majority of task combinations. In summary, the most promising combination of tasks in a 4-class BCI were (1) motor imagery of the left hand (2), one brain-teaser task (word association or mental subtraction) (3), mental rotation task, and (4) one more dynamic motor or non-motor imagery task (auditory imagery, spatial navigation, imagery of the feet).

For the selected task combination, the frequency range within 8-30 Hz that yielded the highest geometric mean was selected individually for online control. As can be seen in Table 1, the lower border of the frequency range was for all users in the alpha band between 8-10 Hz in the screening and between 8-11 Hz in the updates. The upper border was not as narrow and varied in the beta range between 13 to 30 Hz between users.

Figure 2 shows the online performance with the number and time of updates as well as the session from which the data was used for the recalculation of the classifier. All users had one update after the sessions 4-6. Three users had a second update after session 8. Except of for user B, the updates were made with data recorded in the last or second to the last session. For 6 out of 8 users, every update increased performance in the following session. For user D, the first update did not immediately increase accuracy, however, a performance increase was seen in later sessions and after the second update. User E did not show an increase in performance. To analyze if an update increased online performance significantly, we averaged the online performance of two sessions before (Mean (M) = 58, Standard Error (SE) = 4.8) and after (M = 68, SE = 4.4) each update, and compared these two means with a paired t-test. Performance was significantly higher after than before an update (t_10_ = 3.1, p < 0.05). To show that these significant changes are due to the update and not only a training effect, we made the exact same analyses with data from all four consecutive sessions that had no update in-between. No significant changes were found (M_before_ = 64, SE = 4.5, M_after_ = 62, SE = 4.1, p = n.s.).

Online performance averaged over all sessions and tasks ranged between 44-84% (SE = 3-5) for the participants (see Table 1). All users managed to control all 4-classes above chance and achieved online accuracies between 58-93% in their best single-sessions (see Table 1). User C achieved a performance of 93% in session 7 while yielding accuracies above 80% in all single-classes. From session 5 on, all users performed better than chance in every session. Accuracy values ≥ 31% are considered significantly above chance level. This was calculated according to the adjusted Wald confidence interval on the 5%-alpha level, which takes the number of classes and trials into account (p ≥ 0.25 ± 0.055 [43]).

### H2. Training effects

The mean online performance showed a linear increase over sessions in 6 users (see Fig. 2). User E and H showed neither an increase nor a decrease in performance over the sessions. Averaged over users and tasks, online performance was significantly higher in the last (M = 70, SE = 4.4) than in the first (M = 58, SE = 6.8) feedback session (paired t-test, t_7_, = 3.1, p < 0.05) and showed a significant linear increase over sessions (linear regression, R^2^ = 0.7, F_1,7_ = 18.5, p < 0.01).

### H3. Temporal components

**Figure 3:**
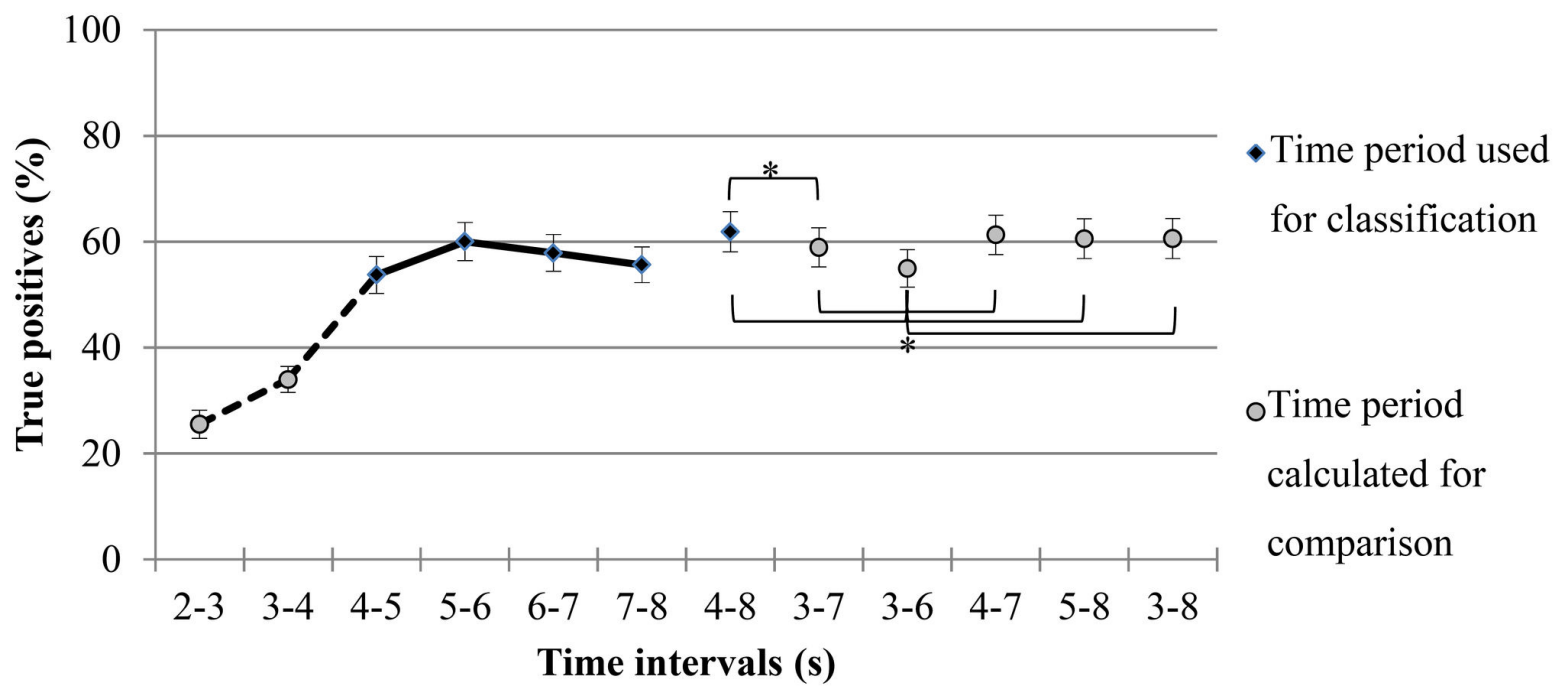
True positives as function of time. The figure shows averaged values over users, sessions and tasks with the standard errors. Left side: The imagery period of t = 2-8 s was classified second-wise. The solid line, which connects the black rhombi, indicates the used classification period (t = 4-8 s after trial onset). Right side: The mean classification accuracies for all possible 3-s, 4-s and 5-s time intervals between t = 3-8 s are shown. The black rhombus shows the used classification period. The grey dots represent other classification time periods for comparison. Significant differences between these time intervals are indicated with an asterisk.

The relevant time for classification was restricted to t = 4-8 s after trial onset (i.e. classification period) within the imagery period (t = 2-8 s after trial onset; cue at t = 2 s). For every second of the imagery period, we calculated the percentage of true positives for each task. In general, the percentage of true positives was low (i.e. random) at the beginning of the imagery period, then increased considerably and decreased slightly again at the end of the trial (see Figure 3). All tasks had their maximum true positive rate within the time period of t = 4-8 s after trial onset. The WORD and AUD tasks had their mean maximum true positive rate between t = 6-8 s. Both tasks showed a rather flat curve and yielded lowest accuracy of all tasks within the classification period of t = 4-8 s. All other tasks peaked within 5-6 s after trial onset.

To evaluate if the classification period can be reduced without a decrease in accuracy, we compared the true positive rate of all possible 3-s, 4-s and 5-s classification intervals within the feedback period from t = 3-8 s by means of an ANOVA (dependent variable: true positive rate, independent variable: time intervals with 6 levels: 4-8, 3-7, 3-6, 4-7, 5-8, 3-8 s). The time interval of t = 3-6 s demonstrated a significantly lower true positive rate than all other time intervals and the time interval of t = 3-7 s showed a significantly lower true positive rate than the time interval of t = 4-8 s (F_1.4, 9.5_ = 15.6, p < 0.01) (see Figure 3, significant results are indicated with an asterisk).

### H4. Feedback

**Figure 4:**
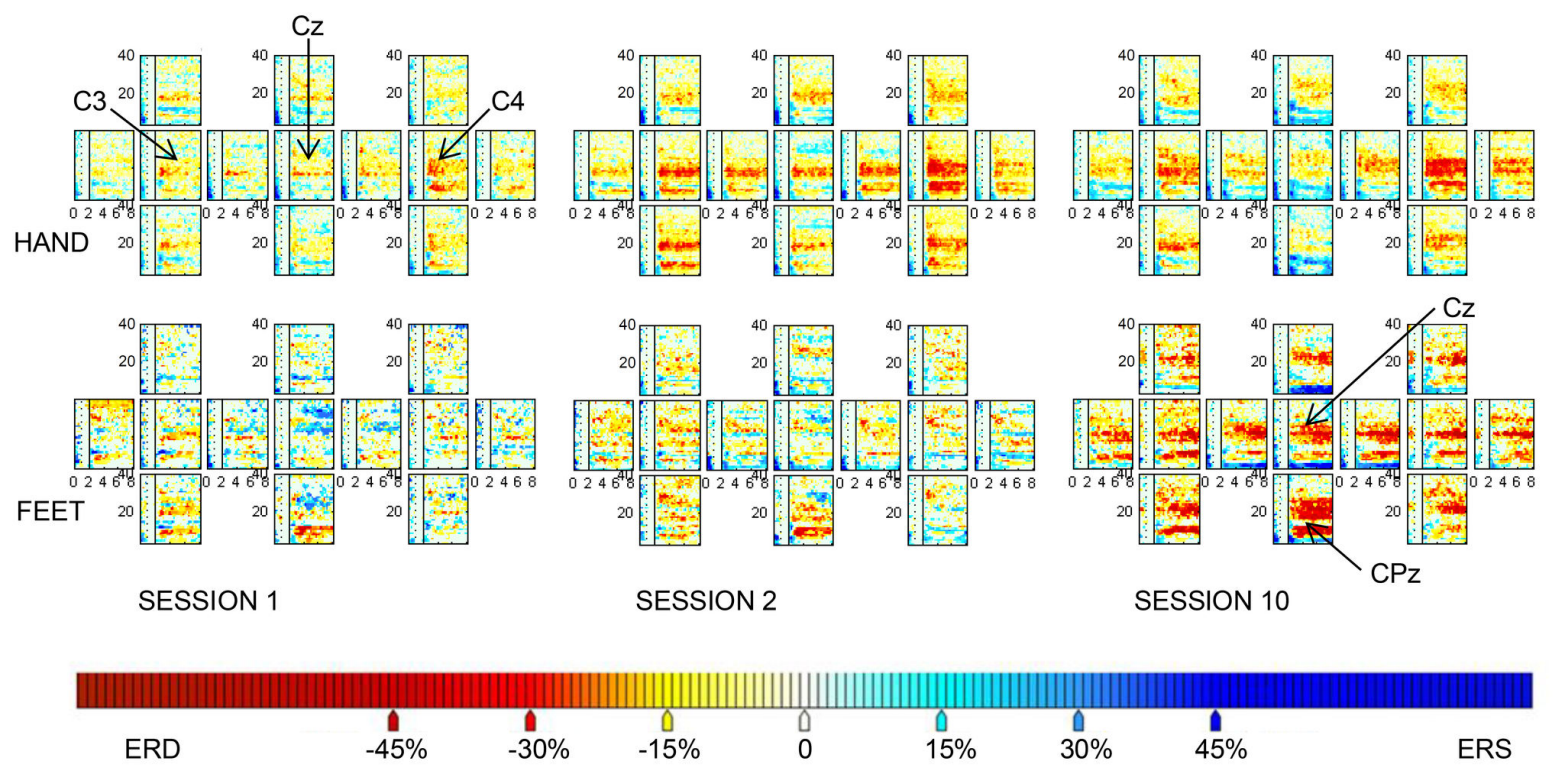
ERD/S patterns. The significant ERD/S patterns are shown for the tasks HAND and FEET for session 1 (screening) and the sessions 2 and 10, both with feedback. Seven users had the task HAND and three users the task FEET in their 4-class combination (see Table 1). Thus, the ERD/S patterns for HAND were averaged over seven users (users A, B, C, D, E, G, H) and the patterns for FEET over three users (users A, C, F). Each pattern includes 13 small maps, which represent the electrode positions FC3, FCz, FC4, C5, C3, C1, Cz, C2, C4, C6, CP3, CPz, CP4. We chose to show only these electrode positions as they are most important for motor imagery tasks and all relevant activation was at these sites. The x-axes indicate the time of a trial (t = 0-8 s, cue at t = 2 s). The y-axes indicate the frequencies (4-40 Hz). A percentage decrease in amplitude in the imagery period (t = 2-8 s after trial onset) relative to the baseline (t = 1-2 s after trial onset; indicated by dashed lines) is indicated in red (ERD, i.e. activation), whereas a percentage increase in amplitude is indicated in blue (ERS). The upper panel shows that the HAND task elicited a contralateral activation at C4 in all sessions. In comparison to the screening session (i.e. session 1), the first feedback session (i.e. session 2) showed an increase in activation as well as more widespread activation. In the course of sessions, the activation became more focused again. In the FEET task (lower panel), most prominent activation could be seen at CPz. The activation hardly increased from the screening to the first feedback session, however, increased substantially in session 10 in comparison to session 2.

Sham feedback was introduced in the screening and calibration runs in order to reduce differences in brain patterns between sessions with and without feedback that are due to visual stimuli. We compared the mean percentage of significant ERD/S values over tasks and electrodes between 8-30 Hz within the imagery period between the screening and the first feedback sessions. Neither for the ERD, nor for the ERS values, did we find a significant difference between the screening and first feedback session (paired t-test, t_7(ERD)_ = 1.8, t_7 (ERS)_, = 0.2, p = n.s.). However, user- and task-specific changes over sessions were also evident. For example, the ERD/S map of the HAND task revealed a considerable difference between screening and feedback sessions (see Figure 4 upper panel). In contrast, in the FEET task, the activation hardly changed between the screening and the first feedback session (see Figure 4, lower panel).

## Discussion and Conclusions

The present protocol aimed to enhance BCI performance by individual optimization of task combinations, frequency range and classifier adaptation, to make selections faster and to reduce the differences in brain patterns between sessions with and without feedback.

Firstly, online accuracies (i.e. H1) and training effects (i.e. H2) were substantially improved with this user-centered protocol in comparison to our previous study in which less individual adjustments were made [23] (see Table 2). In our previous study, the performance stayed stable over multiple months, however, no increase in performance could be shown over the training sessions [23]. In contrast, a significant increase in performance over sessions was shown in the present study. Although there was still some variability in the classification accuracy between tasks and sessions, the individual classifier updates proved to increase performance significantly.

**Table 2:** Comparison of the online performance achieved in this study to our previous study [23].

	Mean performance [%] over sessions (SE)	Peak performance [%] in single-sessions	Accuracy of all 4 tasks significantly above chance in single-sessions
Present study	44-84 (3-5)	58-93	8 out of 8 users
Previous Study [23]	28-64 (5-7)	38-72	8 out of 14 users

The range of mean performance averaged over sessions and tasks with standard error (SE) and of the peak performance in single-sessions achieved by all users is indicated. Eight out of 8 users performed significantly above chance in all tasks during single-sessions in the present study, whereas 8 out of 14 participants did so in the previous study.

The performance achieved in the present protocol was also comparable to results from other motor imagery-based 2D BCIs (e.g. [44]). In the present protocol, users achieved control in fewer sessions. However, controlling a cursor in the 2D space included more target positions and thus was more complex than controlling the cursor only in 4 directions. For real-world applications, however, BCIs based on event-related potentials, such as the P300 speller, are mostly used to date [45]. The P300 can be detected in 90% of people with minimal training required which makes the P300 speller highly effective [45,46]. Depending on the specific method and application used, comparably high or higher accuracies and information transfer rates were achieved with P300 spellers than in the present study [47,48]. However, users must always rely on an external cue from the system when using an event-related potential-based BCI. In contrast, mental-imagery BCIs are based on the voluntary modulation of oscillatory components and can therefore be used in self-paced protocols which is an advantage for real-world applications and our ultimate goal as they provide on-demand access to communication [1,14].

Typically, about 15–30% of participants are not able to control a mental-imagery based BCI system [46,49]. In the present study, all users performed significantly above chance from session 5 on. This indicates that the proper selection of control strategies and system parameters, as proposed by our protocol, facilitates users in getting control of a BCI, at least at a basic level. Thus, this protocol could increase the percentage of users who gain and maintain BCI control. Although our results are very promising, the sample of participants in the present study is not large enough to make sound claims for the population.

Based on these results, our first and second hypotheses can be accepted: The individual adjustments (H1) enabled all users to perform significantly above chance and (H2) led to a significant increase in performance over sessions. The results also confirmed that the best mental tasks for BCI control are highly-individual specific [13,19–21]. However, a pre-selection of reliable and robust mental tasks from which the users can select is very important, as a screening with many mental tasks is very time consuming and exhausting for participants [46]. Therefore, some general conclusions should be drawn from this study. Motor imagery of the hand was most often included in the task combinations and demonstrated high performance as shown in previous findings [23,27] (see Table 1). Our results are also in line with Blankertz et al. [31], who reported good discriminability between two motor imagery tasks around 10 Hz which extended up to the higher beta band for the majority of users. Besides motor imagery, every user had one but never two brain-teaser tasks included in this study. This supports a combined use of brain-teaser and dynamic imagery tasks [18] and opposes the use of two brain teaser tasks in one paradigm [23]. Although the mental rotation task showed low temporal stability of the ERD/S patterns and contradictory offline classification results in previous studies [18,28], it revealed good performance in the present protocol. These findings suggest that, although offline results build an important basis for research, only real-time experiments can confirm whether given methods will work for online control. To summarize, a combination of (1) motor imagery of the left hand, (2) one brain-teaser task (word association or mental subtraction), (3) mental rotation task, and (4) one more dynamic motor or non-motor task (auditory imagery, spatial navigation, imagery of the feet) seems to be the best choice for able-bodied users with the present BCI protocol.

Secondly, the temporal evolution (i.e. H3) of classification was considered. Previous cue-based BCIs used relatively short imagery periods of t = 1.5-7 s for motor imagery tasks (e.g. [10,23,50,51]) and relatively long imagery periods of t = 7-10 s for non-motor tasks (e.g. [15,16,23]). In the present study, the imagery period was restricted to t = 6 s (t = 2-8 s after trial onset) and the classification period to t = 4 s (t = 4-8 s after trial onset) which made shorter trials and thus faster selections possible as compared to previous cue-based protocols including non-motor tasks [15,16,23]. Users were able to show sustained brain patterns for a period of 4 s, starting 2 s after the cue onset which was long enough to classify four different mental tasks in this BCI protocol (see Figure 3). These findings are in line with simulated time courses of pair-wise classification of different mental tasks [3] as well as with motor imagery paradigms [10,50].

Our offline analyses indicated that a 4-s classification period does not decrease performance in comparison to a 5-s classification period (see Figure 3). Thus, we can accept our third hypotheses that classification can be restricted to 4-s starting 2 s after the cue without a decrease in performance. The comparably high classification results of the 3-s classification period indicate that a further restriction of the imagery period to t = 2-7 s and classification period to t = 4-7 s might be possible without a substantial decrease in performance. Furthermore, individualized time intervals for tasks as well as users might further increase accuracy and speed.

Thirdly, we introduced sham feedback - to mimic real-time feedback - in the screening and calibration runs in which usually no continuous feedback is displayed. We did this to minimize the impact of changing visual information due to continuous feedback between sessions [10,23,32,36] (i.e. H4). In our previous study [23], activation increased considerably in the feedback sessions in comparison to the screening sessions in all tasks. Neuper et al. also found significant differences between the screening and feedback sessions in the ERD values [10]. In contrast, no significant differences could be found in the mean percentage of ERD/S values between screening and feedback sessions in the present study. This suggests that the sham feedback in the screening sessions contributed to reduce the differences between screening and feedback sessions. However, task- and user-specific differences could be observed. Therefore, this result needs to be replicated with a greater sample size in future studies which allows taking task-specific differences into account in order to accept our fourth hypothesis. As the sham feedback has a different meaning to the users than the real-time feedback, some changes in brain patterns can always be expected.

In summary, the present study demonstrated design solutions to meet the user requirement for more accurate and faster BCI control [24,25]. Although our results are promising, further improvements in the overall accuracy and speed as well as in other aspects of an user-centered approach are necessary before this protocol reaches the level of practical use and can be applied independently and comfortably in one’s every-day life [24]. A high priority for future work is to evaluate the present BCI protocol with severely motor impaired individuals. Their impairment may be directly responsible for decreases in performance of certain mental tasks [52] or be associated with other neurological or attentional deficits that make it difficult to perform the tasks. Therefore, results from able-bodied persons cannot necessarily be generalized to disabled individuals [53]. Besides visual feedback, other feedback modalities should be incorporated and BCI applications should be adapted to each individual’s special needs [46,53]. Furthermore, the selection of mental tasks should be based not only on classification results but also on the user’s personal preferences for specific mental tasks [22]. Thus, performance as well as user-comfort could be enhanced.

To conclude, the present protocol improved 4-class BCI performance significantly by individual selection of control strategies and frequency range while making selections faster in comparison to previous cue-based protocols using non-motor tasks. Furthermore, classifier adaptation was optimized individually and the differences in brain patterns between sessions with and without feedback could be reduced. This protocol is highly individual adjustable and could increase the percentage of users who gain and maintain BCI control.
